# Increased UHMWPE Particle-Induced Osteolysis in Fetuin-A-Deficient Mice

**DOI:** 10.3390/jfb14010030

**Published:** 2023-01-04

**Authors:** Christina Polan, Christina Brenner, Monika Herten, Gero Hilken, Florian Grabellus, Heinz-Lothar Meyer, Manuel Burggraf, Marcel Dudda, Willi Jahnen-Dechent, Christian Wedemeyer, Max Daniel Kauther

**Affiliations:** 1 Department of Trauma, Hand and Reconstructive Surgery, University Hospital Essen, University Duisburg-Essen, 45147 Essen, Germany; 2 Central Animal Laboratory, University Hospital Essen, University Duisburg-Essen, 45147 Essen, Germany; 3 Institute of Pathology and Neuropathology, University Hospital Essen, University Duisburg-Essen, 45147 Essen, Germany; 4 Helmholtz-Institute for Biomedical Engineering, RWTH Aachen University Hospital, 52074 Aachen, Germany; 5 Department of Orthopaedic Surgery, St. Barbara Hospital Gladbeck, 45964 Gladbeck, Germany; 6 Department of Trauma Surgery and Orthopedics, Pediatric Orthopedics, Agaplesion Diakonieklinikum Rotenburg (Wümme), 27356 Rotenburg, Germany

**Keywords:** implants, arthroplasty, aseptic prosthetic loosening, osteolysis, particle-induced osteolysis, mouse calvaria osteolysis model, polyethylene particles, wear particles

## Abstract

Particle-induced osteolysis is a major cause of aseptic prosthetic loosening. Implant wear particles stimulate tissue macrophages inducing an aseptic inflammatory reaction, which ultimately results in bone loss. Fetuin-A is a key regulator of calcified matrix metabolism and an acute phase protein. We studied the influence of fetuin-A on particle-induced osteolysis in an established mouse model using fetuin-A-deficient mice. Ten fetuin-A-deficient (*Ahsg^−/−^*) mice and ten wild-type animals (*Ahsg^+/+^*) were assigned to test group receiving ultra-high molecular weight polyethylene (UHMWPE) particle implantation or to control group (sham surgery). After 14 days, bone metabolism parameters RANKL, osteoprotegerin (OPG), osteocalcin (OC), alkaline phosphatase (ALP), calcium, phosphate, and desoxypyridinoline (DPD) were examined. Bone volume was determined by microcomputed tomography (μCT); osteolytic regions and osteoclasts were histomorphometrically analyzed. After particle treatment, bone resorption was significantly increased in *Ahsg^−/−^* mice compared with corresponding *Ahsg^+/+^* wild-type mice (*p* = 0.007). Eroded surface areas in *Ahsg^−/−^* mice were significantly increased (*p* = 0.002) compared with *Ahsg*^+/+^ mice, as well as the number of osteoclasts compared with control (*p* = 0.039). Fetuin-A deficiency revealed increased OPG (*p* = 0.002), and decreased levels of DPD (*p* = 0.038), OC (*p* = 0.036), ALP (*p* < 0.001), and Ca (*p* = 0.001) compared with wild-type animals. Under osteolytic conditions in *Ahsg^−/−^* mice, OPG was increased (*p* = 0.013), ALP (*p* = 0.015) and DPD (*p* = 0.012) were decreased compared with the *Ahsg^+/+^* group. Osteolytic conditions lead to greater bone loss in fetuin-A-deficient mice compared with wild-type mice. Reduced fetuin-A serum levels may be a risk factor for particle-induced osteolysis while the protective effect of fetuin-A might be a future pathway for prophylaxis and treatment.

## 1. Introduction

The aseptic osteolysis, or particle disease, is the major cause of long-term failure of arthroplasty [1] and is evoked by wear debris particles generated by shear and friction forces. Wear particles from prosthesis components such as ultra-high molecular weight polyethylene (UHMWPE), titanium, ceramic, or cement particles are phagocytosed by macrophages, which subsequently trigger an inflammatory cascade via the release of proinflammatory cytokines, leading to loss of bone substance and loosening of endoprosthesis [2,3]. The inflammatory reaction depends on the number, size, material, and shape of the abrasive material. The inflammatory reaction increases with the particle concentration [4,5].

Bone tissue, which consists of minerals such as calcium and phosphate in the form of hydroxyapatite organic material, undergoes constant modification. An imbalance of the bone formation and degradation in favor of bone loss leads to various pathologies such as, among other things, the aseptic osteolysis in prostheses. In addition to numerous other cells, such as stem cells or immune cells, bone tissue consists of bone-degrading osteoclasts, bone-building osteoblasts, and osteocytes [6]. Bone resorption is regulated by the release of messenger substances, hormones, and cytokines, which regulate the differentiation and activation of osteoclasts.

Key regulator of bone metabolism is the RANK-RANKL-OPG system, which consists of the messenger substances osteoprotegerin (OPG), the ligand of the receptor activator of the nuclear factor κB (RANKL) and the associated receptor (RANK) [7,8,9]. The ratio of bone-protective OPG to bone-destructive RANKL determines the balance between bone formation and bone resorption that is imbalanced in aseptic loosening [3,10]. Macrophages can increase RANKL release through the secretion of proinflammatory cytokines such as IL-1β and TNF-α and promote bone resorption by stimulating osteoclast function [11,12,13].

Fetuin-A (alpha2-Heremans-Schmid glycoprotein (Ahsg)) is highly enriched in the mineralized bone matrix [14,15]. Fetuin-A plays an essential role in the solubility of serum calcium, inhibits the precipitation of calcium through the formation of calcium-protein complexes and influences the formation of mineralized bone [16,17,18,19]. A recent study suggested that fetuin-A is a multifaceted protective factor that locally counteracts calcification, modulates macrophage polarization, and attenuates inflammation and fibrosis, thus preserving tissue function [20].

The majority of publications support that fetuin-A acts as an indirect anti-inflammatory agent [20,21]. Fetuin-A is known as negative acute phase protein in injury and infection [22,23,24]. A recent review concluded that fetuin-A is down-regulated in degenerative joint disease and can be used as a biomarker in the diagnosis and treatment of arthritis [25]. In a murine calvaria model of particle-induced osteolysis, a single dose of fetuin-A minimized bone resorption under osteolytic conditions demonstrating a protective effect of Fetuin-A [26]. This calvaria model is an established animal model for the investigation of particle-induced osteolysis [27,28]. Following peri-implant osteolysis in human joint endoprostheses, polyethylene particles are applied in a very high concentration to the cranial calotte of the calvaria model. This results in an aseptic inflammatory reaction, the formation of granulation tissue rich in macrophages, and a change in local bone metabolism with the activation of osteoclasts.

Given the pleiotropic effects of fetuin-A in bone metabolism, the question of whether fetuin-A deficiency might also play a role in particle-induced osteolysis prompted us to further investigate the influence of fetuin-A on particle-induced osteolysis in an established mouse model of aseptic loosening using homozygot wild-type (*Ahsg^+/+^*) and homozygot fetuin-A-deficient (*Ahsg^−/−^*) mice. Our hypothesis was that in absence of fetuin-A the particle-induced inflammatory response and osteolysis is increased.

## 2. Materials and Methods

### 2.1. Animals and Ethics

The animal experiment was carried out in accordance with the international regulations for use and care of laboratory animals and the reporting of in vivo experiments (ARRIVE) guidelines and was approved by the national district government (State Office for Nature, Environment and Consumer Protection of North Rhine-Westphalia, LANUV AZ 9.93.2.10.34.07.138). Ten 12-week-old male fetuin-A-deficient mice (*Ahsg^−/−^*, ILAR strain BL6, Ahsgtm1, wja, originated from backcrossing of the original C57BL/6-129/Sv hybrid mice) as well as ten age- and gender-matching wild-type *Ahsg^+/+^* (wt) mice of the C57 Bl 6/N strain (Charles River Wiga GmbH, Sulzfeld, Germany) were used [29,30]. The specific pathogen-free animals were kept in cages in a climate-controlled room (22 °C; 45–54% relative humidity) with a 12 h light/dark cycle in groups until surgery and as single animals after surgery. The cages were equipped with bedding, nesting material, and shelter (spacious plastic house). Food and water were allowed ad libitum. The animals were monitored on a daily interval. In case of any abnormality, the individual stress of the animal concerned was evaluated with an experiment-specific score sheet. On the first and last experimental days, the animals were housed in metabolic cages for urine collection.

### 2.2. Ultra-High Molecular Weight Polyethylene UHMWPE Particles

The Clariant Company (Gersthofen, Germany) supplied the UHMWPE polyethylene particles (Ceridust VP 3610). More than 35% of the particles were smaller than 1 µm showing a mean particle size (provided as equivalent circle diameter) of 1.75 ± 1.43 µm (range 0.05–11.6 µm). The surface of the particles was rougher, and the particles were shorter in length, displaying a more uniform size distribution in relation to particles obtained in hip joint simulators. To remove putative endotoxins and for disinfection, the particles were washed twice in 70% ethanol at room temperature for 24 h, followed by washing in sterile phosphate buffered saline and drying [31]. The endotoxin content of the particles was controlled (Limulus Amebocyte Lysate (LAL) Assay) to be lower than the detection level of <0.25 EU/mL.

### 2.3. Animal Surgery

**Figure 1:**
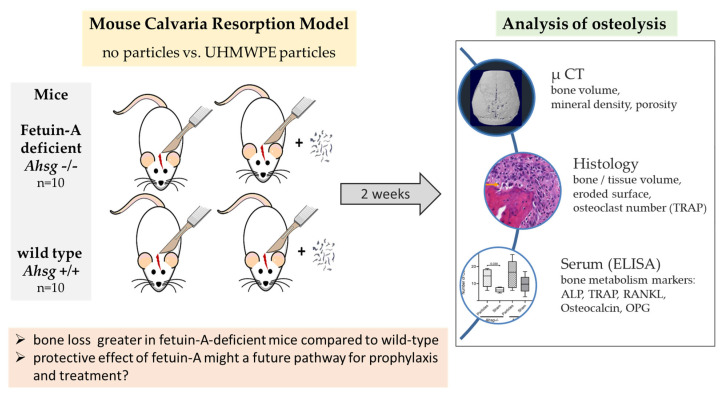
Graphical abstract illustrating the different group distribution as well as the applied methods.

Ten *Ahsg^−/−^* mice and ten wt mice were randomly assigned to either particle implantation or a sham operation (*n* = 5 per group and type) (see graphical abstract Figure 1). An anesthetic mixture of ketamine and xylazine (CEVA Tiergesundheit, Düsseldorf, Germany) was administered intraperitoneally before the animals were anesthetized with isoflurane followed by an intraperitoneal injection of 0.1 g/kg bodyweight (BW) ketamin/0.01 g/kg BW xylazine in NaCl. Before surgery, 300 µL blood was drawn by orbital puncture. A 10 mm incision was made along the calvarial sagittal midline suture exposing an area of 10 mm × 10 mm in the periosteum. The animals in the particle groups received a total volume of 30 µL dried UHMWPE particles (corresponding to 6 × 10^6^ particles), which were distributed over the periosteum using a sterile sharp surgical spoon as previously described [32]. In the sham controls, the incision was closed without any further intervention.

Subsequently, each animal received buprenorphine (0.1 mg/kg BW, Temgesic^®^, Reckitt Benckiser, Slough, UK) for postoperative analgesia.

Fourteen days after operation, the animals were sacrificed using CO_2_ aspiration, and blood samples were taken immediately by cardiac puncture. The skulls were removed and fixed in 10% formalin. Apart from the conduction of the surgery, further analyses (outcome assessment and data analysis) were performed in a blinded condition.

### 2.4. Microcomputed Tomography

The radiological examination of the skulls was performed by microcomputer tomograph (µCT) (X-ray microcomputer tomograph 1072, Skyscan, Aartselaar, Belgium). Two-dimensional X-ray images were realized using the TomoNT program (Skyscan) with a 19.23× magnification for the images. The X-ray source was set to a voltage of 80 kV at an amperage of 100 µA. At an exposure time of 4.9 s and a rotation angle of 0.9° per image, a total angle of 180° was scanned. A field correction was also activated. The greyscales were selected so that the tissue was radiographically displayed mainly around the bone window. The cone-beam reconstruction program generated a total of about 700 sectional images. A quantitative analysis of bone volumes and bone surfaces was performed using a CT-analysis program (CTAn, Skyscan). Five defined cuboids (8 mm^3^) along the sagittal suture were selected for investigation of the following parameters: bone volume (BV), bone surface (BS), and bone volume per tissue volume (BV/TV). Subsequently, a three-dimensional model of the calvaria was created (CTVol Surface Rendering, Skyscan).

### 2.5. Bone Histomorphometry

The calvaria were prepared from the skulls and decalcified for seven days (Osteosoft^®^, Merck, Darmstadt, Germany) and embedded in paraffin. Sectional images of 4 µm thickness were prepared and stained with hematoxylin and eosin (HE) to determine the area of bone resorption in the midline suture. Additionally, the osteoclasts were identified as large multinucleated tartrate resistant alkaline phosphatase (TRAP) positive cells (Sigma Aldrich, Deisenhofen, Germany). The area of bone resorption containing the osteolytic areas was defined as eroded surface (ES) and measured with special software (Image Tool 3.0, University of Texas, San Antonio, TX, USA) [33].

### 2.6. Analyses of the Parameters of Bone Metabolism

Serum was stored at −70 °C until analysis. All assays were performed according to the instructions of the manufacturers. Calcium, phosphate, and ALP were determined using an RX Monza Analyzer (Randox, Antrim, UK). For OPG and RANKL, immunoassays were used (Quantikine assays, R&D Systems, Minneapolis, MN, USA), while OC was analyzed with an immunoradiometric assay (Immutopics, San Clemente, CA, USA). Deoxypyridinoline (DPD), a crosslink product of collagen molecules, was determined in urine samples (Quidel, San Diego, CA, USA). Creatinine was measured in urine samples within two hours after collection using a Siemens ADVIA 2400 analyzer (Siemens Medical Solutions, Fernwald, Germany). Variations in urine concentration were eliminated using the ratio of DPD [nmol/L] and the urine creatinine concentration [mmol/L], resulting in a value without units.

### 2.7. Statistical Analysis

Estimation of the number of cases for changes in bone volume resulted in a case number of 5 with a power of 80% and a probability of error of <0.05%.

The data were analyzed using the statistics program SPSS 27 (IBM, New York, NY, USA). For all continuous parameters, the mean value, standard deviation, minimum, maximum, and median were determined based on descriptive statistics. The individual groups were examined for normal distribution using the Shapiro–Wilk test for small samples. Parametric values were tested for significance using the two-way analysis of variance (ANOVA) to analyze an influence of particles (main effect), group (main effect), or the combination of particles and group (interaction effect). Normally distributed data sets were examined with a two-sided *t*-test for independent samples to study the difference in the means between pairs of groups. For nonparametric values, the Wilcoxon rank-sum test and the Mann–Whitney U test were performed. A *p*-value < 0.05 was considered as significant.

## 3. Results

No surgical or postoperative complications occurred. All animals were euthanized at the end of the follow-up period.

### 3.1. Micro Computed Tomography

**Figure 2:**
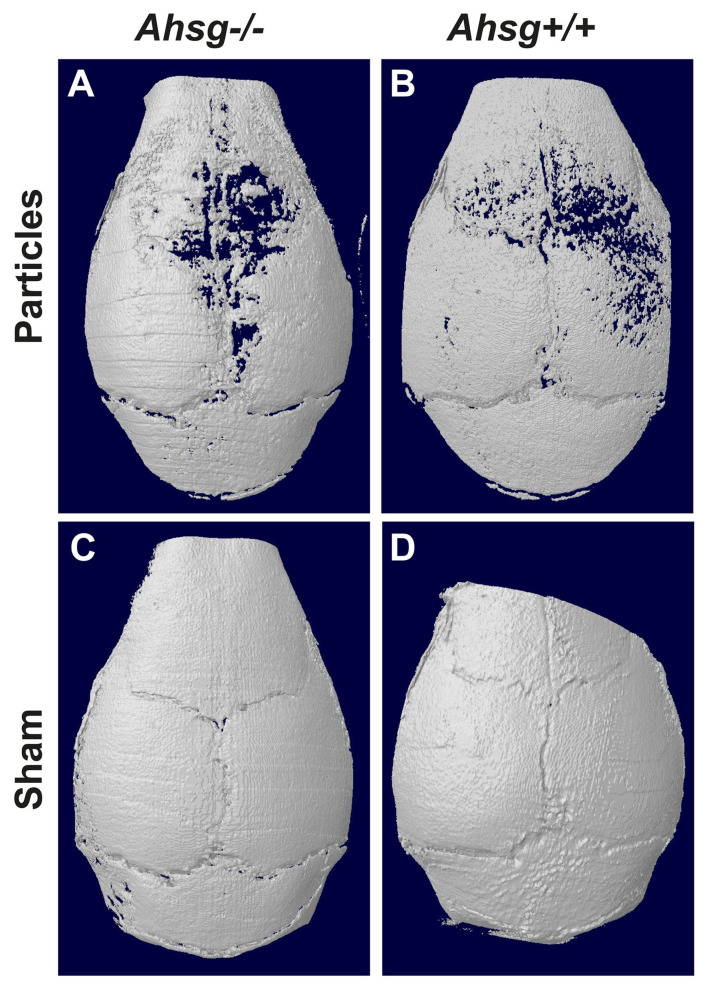
Three-dimensional reconstruction of the calvaria. After 14 days, the calvarial bone was analyzed by µCT. (**A**) Particle treatment in mice with the *Ahsg^−/−^* background led to severe osteolysis. (**B**) In wild-type mice, less osteolysis was seen after particle treatment. (**C**) Sham-operated fetuin-A-deficient mice, and (**D**) sham-operated wild-type mice did not show any signs of osteolysis.

Three-dimensional reconstructions of the calvaria showed the qualitative extent of particle-induced osteolysis (Figure 2). Particle treatment led to bone loss and circular osteolysis, especially close to the sagittal suture where most of the particles were implanted (Figure 2A,B), whereas the sham-operated mice did not have any osteolytic lesions (Figure 2C,D). The osteolytic regions were largest in the fetuin-A-deficient particle-treated group (Figure 2A).

**Figure 3:**
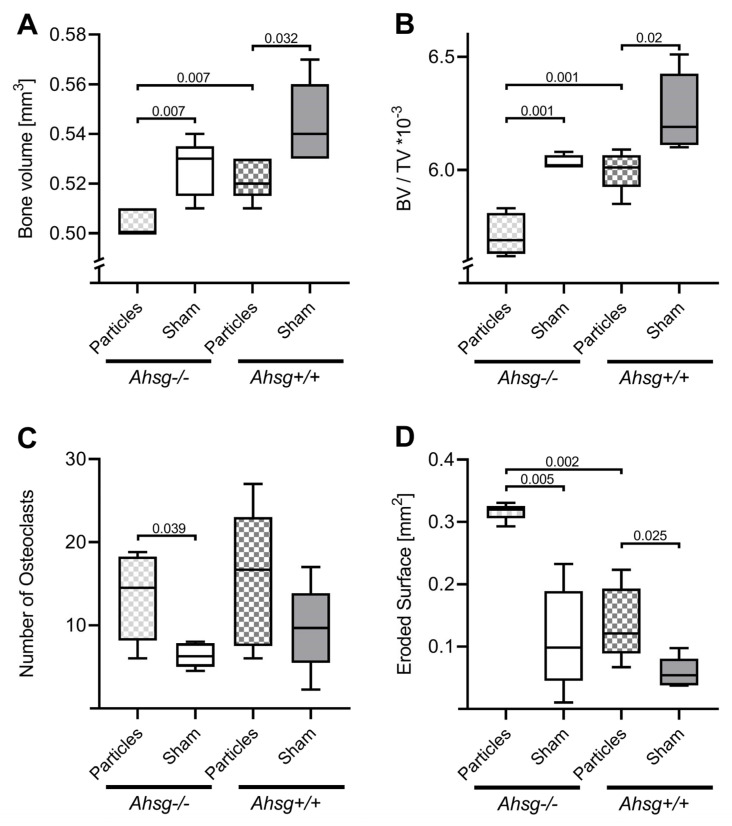
Bone volume and ratio of bone volume/tissue volume analyzed by µCT analysis and number of osteoclasts and eroded surface area at two weeks after surgery as box plots measured in in the histological sections. (**A**) Bone volume. (**B**) Ratio of bone volume/tissue volume. (**C**) Number of osteoclasts. (**D**) Eroded surface area.

The analysis of bone volume in the target region showed an influence of UHMWPE particle implantation on the radiologically quantifiable bone tissue of both genotypes (Figure 3). Osteolytic conditions resulted in a significant decrease in bone volume in the fetuin-A-deficient group (*p* = 0.007) as well as in the wt group (*p* = 0.032) compared with the respective sham-operated groups (Table 1) (Figure 3A). Fetuin-A-deficient animals displayed a significant lower bone volume after particle treatment than wild-type animals (*p* = 0.007). Osteolytic conditions led to a significant reduction in the ratio BV/TV in fetuin-A-deficient mice (*p* = 0.001) as well as in wild-type mice (*p* = 0.02) compared with the respective sham-operated groups. After particle treatment, fetuin-A-deficient mice had a significantly lower BV/TV ratio than wt mice (*p* = 0.001) (Table 2) (Figure 3B). No significant differences were found between the groups regarding the bone surface in µCT (data not shown).

### 3.2. Bone Histomorphometry

**Figure 4:**
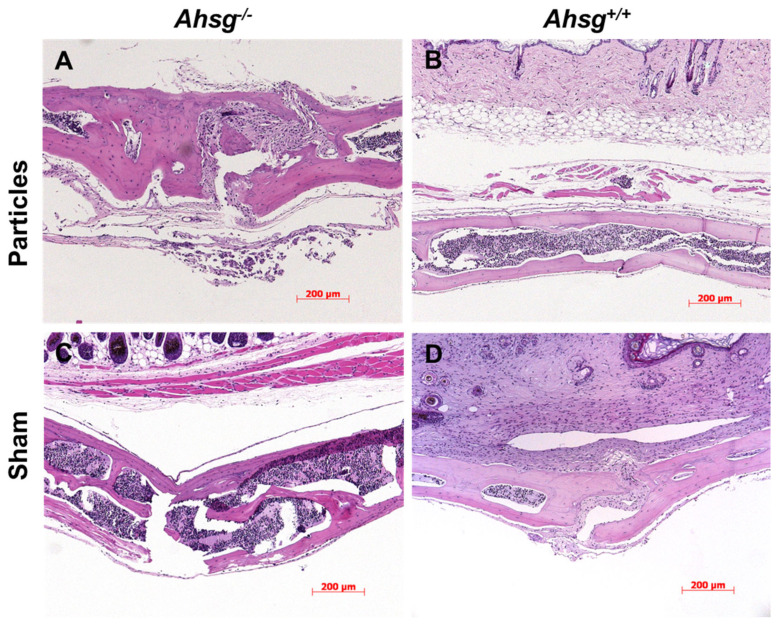
HE staining of calvarial sections. After 14 days, the calvarial bone was fixed, embedded and histologically stained. (**A**) Particle treatment in mice with the *Ahsg^−/−^* background led to a highly eroded surface. (**B**) In wild-type mice, less eroded surface was seen after particle treatment. (**C**) Sham-operated fetuin-A-deficient mice and (**D**) sham-operated wild-type mice did not show any signs of osteolysis. Scale bar indicates 200 µm.

**Figure 5:**
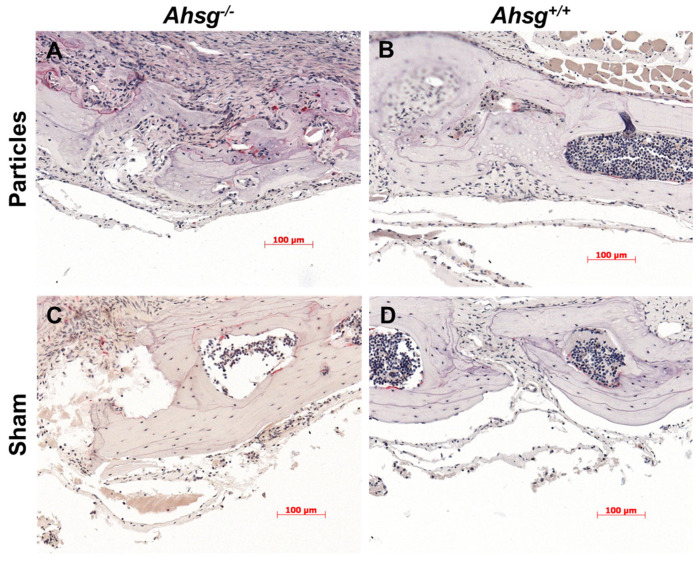
TRAP staining of calvarial sections. After 14 days, the calvarial bone was fixed, embedded and the osteoclast stained by tartrate resistant alkaline phosphatase. Particle treatment in the mice with the *Ahsg^−/−^* background (**A**) and in the wild-type mice (**B**) led to a visible increase in osteoclasts compared with the sham-operated animals (**C**,**D**). Both sham-operated fetuin-A-deficient mice (**C**) and sham-operated wild-type mice (**D**) had fewer osteoclasts. Scale bar indicates 100 µm.

Under osteolytic conditions, the granulation tissue of both groups was infiltrated with macrophages and polynucleated giant cells in the area adjacent to the implanted UHMWPE particles (Figure 4A,B). At the same time, macrophages were found in typical resorption lacunae in the calvarial bone resulting in osteolytic lesions. In the sham groups, granulation tissue was also present but displayed only few inflammatory reactions. TRAP staining revealed an increased number of osteoclasts in both particle-treated groups compared with the sham groups, which was statistically significant for fetuin-A-deficient mice (*p* = 0.039) (Figure 3C and Figure 5), (Table 3).

Histomorphometric investigation of the eroded surface (ES) in the osteolytic areas showed that particle treatment led to a significant increase in ES in both genetic backgrounds, *Ahsg^+/+^* (*p* = 0.025) as well as *Ahsg*^−/−^ (*p* = 0.005) (Figure 3D) (Table 4). Fetuin-A-deficient mice displayed the largest ES compared with wild-type mice under osteolytic conditions (*p* = 0.002). In the sham-operated animals, no difference was detected between the groups.

### 3.3. Parameters of Bone Metabolism

**Figure 6:**
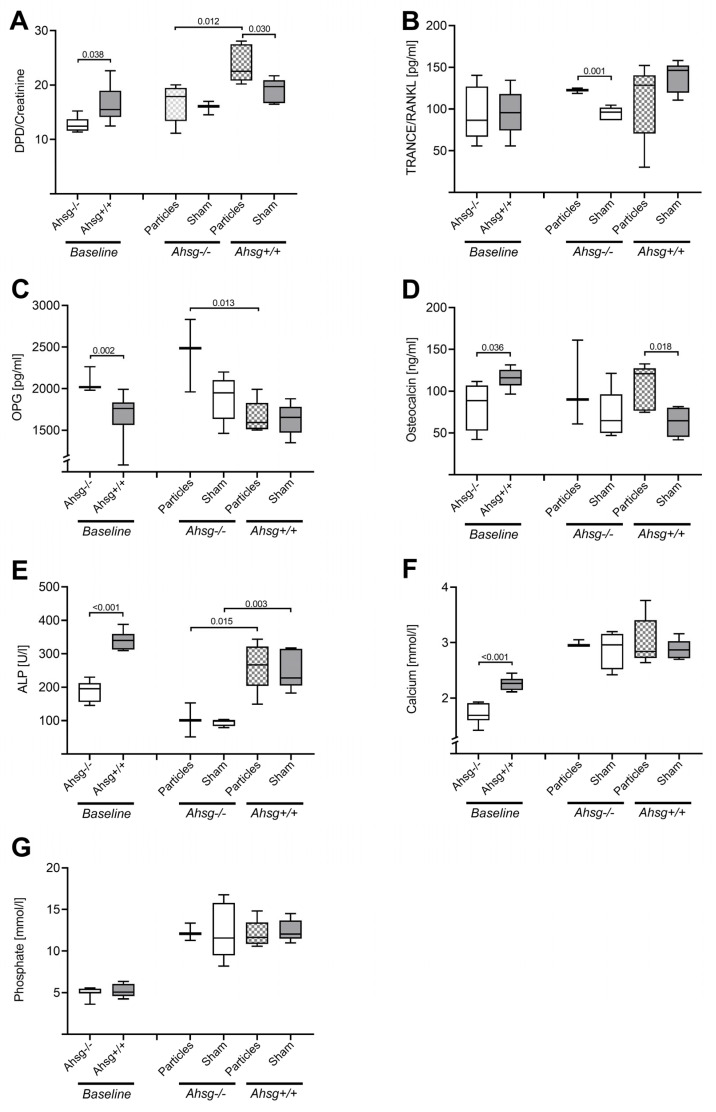
Bone metabolism parameters as box plots. The figure shows the single values of *n* = 5 animals per group with the median as line. *Ahsg^−/−^* = fetuin-A-deficient animals, *Ahsg^+/+^* = wild-type mice. Significant differences between the groups are indicated with *p*-values. (**A**) DPD/Creatinine. (**B**) RANKL. (**C**) Osteoprotegerin (OPG). (**D**) Osteocalcin. (**E**) Alkaline phosphatase (ALP). (**F**) Serum calcium. (**G**) Serum phosphate.

We determined bone- and mineral-related serum chemistry to monitor bone metabolism in *Ahsg^−/−^* and *Ahsg^+/+^* mice before and after particle-induced osteolysis (Figure 6).

Preoperatively, the *Ahsg^−/−^* mice had significantly lower DPD values than the *Ahsg^+/+^* animals (*p* = 0.038) (Table 5) (Figure 6A).

After particle treatment, the *Ahsg^−/−^* mice showed significantly lower DPD concentrations compared with the wild-type animals (*p* = 0.012). Particle addition led to significant higher DPD values for the wt animals (*p* = 0.030) while there were no differences in *Ahsg^−/−^* animals compared with sham control.

The baseline RANKL values of both genetic backgrounds were comparable (Table 5) (Figure 6B). In fetuin-A-deficient mice, particle treatment led to increased RANKL values compared with the sham-operated mice (*p* = 0.001). In osteolytic conditions, the *Ahsg^−/−^* mice showed similar RANKL values compared with the *Ahsg^+/+^* mice. In the wt animals, there was no difference in the RANKL values between the particle-treated and the sham-operated mice.

Preoperative OPG levels were significantly increased in *Ahsg^−/−^* mice (*p* = 0.002) compared with wt animals (Table 5) (Figure 6C). In the postoperative examination under osteolytic conditions, a significantly increased OPG value was detected in the *Ahsg^−/−^* mice in comparison to the *Ahsg^+/+^* mice (*p* = 0.013). There was no statistically significant difference between the particle-treated and the sham-operated mice of either genetic background.

Baseline levels of osteocalcin revealed significantly lower values in the fetuin-A-knockout animals compared with the wild-type mice (*p* = 0.036) (Table 5) (Figure 6D). Particle treatment resulted in significantly higher OC concentrations in the wild-type mice compared with sham-operated animals (*p* = 0.018). The other groups did not differ significantly.

The fetuin-A-deficient mice had significantly lower systemic alkaline phosphatase levels compared with the wild-type mice (*p* < 0.001) (Table 5) (Figure 6E). *Ahsg^−/−^* mice had significantly decreased ALP activity compared with wt animals in both treatment groups, after particle treatment (*p* = 0.015) and in the sham animals (*p* = 0.003).

Before surgery, the fetuin-A-deficient animals had a lower serum calcium level than the wild-type mice (*p* < 0.001) (Table 5) (Figure 6F). Postoperatively, no significant differences in calcium level were detected between the subgroups (genetic background and treatment).

The baseline phosphate levels did not differ between the genetic background groups (Table 5) (Figure 6G). Likewise, postoperative scores also did not differ between the different genetic background groups or between the different surgical groups.

## 4. Discussion

### 4.1. In Absence of Fetuin-A, the Particle-Induced Inflammatory Response and Osteolysis Is Increased

A previous study suggested a possible attenuating role of the hepatic serum protein fetuin-A a key regulator of calcified matrix metabolism, and particle-induced osteolysis [26].

Our hypothesis, that in absence of fetuin-A the particle-induced inflammatory response and osteolysis is increased, was confirmed. After particle treatment and comparison with wild-type mice, in fetuin-A-deficient mice the bone volume and the ratio of bone volume to tissue volume was significantly decreased while the eroded surface area was significantly increased. Additionally, the number of osteoclasts was significantly increased compared with sham animals. These results provide novel insights demonstrating that bone resorption was significantly increased in fetuin-A-deficient mice compared with wild-type mice under osteolytic conditions. This was confirmed by significant changes in bone metabolism markers compared with wild-type animals in fetuin-A-deficient animal: OPG was increased, DPD, Ca, ALP, and OC were decreased.

### 4.2. Comparing the Phenotypes before Particle Treatment: Fetuin-A-Deficient Mice Presented Differences Regarding Bone Micro-Structure in µ-CT and Serum Analytes

In our study, the phenotype of wild-type mice and fetuin-A-deficient mice maintained against the genetic background C57BL/6 presented differences regarding bone micro-structure in µ-CT and serum analytes. The µCT analysis presented reduced BV/TV in the *Ahsg^−/−^* mice, which is in line with the findings of Schäfer et al. [16]. The calcium levels in the *Ahsg^−/−^* mice were lower than those in the wild-type and the *Ahsg^−/−^* mice with a DBA/2 genetic background described by Schäfer et al. [16]. This emphasizes the importance of using mice with matching genetic backgrounds, as was performed in this study. The phenotype of *Ahsg^−/−^* under osteolytic conditions was accentuated by increased OPG levels in the present study. Lower levels of alkaline phosphatase activity in *Ahsg^−/−^* mice might relate to the genetic background and were not influenced by particle stimulation.

### 4.3. Potential Mechanisms of Fetuin-A in Particle Treatment: Fetuin-A Attenuates UHMWPE Particle-Induced Local Inflammation by Aiding the Removal of Calcified Cellular and Tissue Debris

We used a standardized model of UHMWPE particle treatment to simulate the effect of particles generated by wear and corrosion of joint replacement prostheses in wild-type and *Ahsg^−/−^* mice. The wild-type mice developed significantly increased osteolysis after UHMWPE particle treatment indicating the validity of the calvarial model as used in previous studies [28]. The fetuin-A-deficient mice showed increased particle-induced osteolysis compared with the wild-type mice, a hitherto unknown influence of the glycoprotein.

Established facts as the role of fetuin-A in mineralized matrix metabolism explains the increase in particle-induced osteolysis in *Ahsg^−/−^* mice. Fetuin-A attenuates UHMWPE particle-induced local inflammation by aiding the removal of calcified cellular and tissue debris. In the absence of fetuin-A such debris triggers a vicious cycle of defective debris clearance and inflammation, and therefore of increased cell death and decreased wound healing and osteogenesis [17]. In addition to aiding calcified debris removal, fetuin-A can exercise an anti-inflammatory action through associated anti-inflammatory polyanions and TGF-ß, as well as though neutralization of HMGB1, a late mediator of cell-damage associated systemic inflammation [17,24,34,35]. Our histological findings of pronounced inflammatory granulomatous tissue on the calvariae of fetuin-A-deficient mice after particle implantation underscore this hypothesis. We propose that the anti-inflammatory action of fetuin-A is due to the mobilization and removal of potentially pro-inflammatory debris, and may be further enhanced by fetuin-A-associated anti-inflammatory agents collectively neutralizing the pro-inflammatory influence of danger associated intracellular molecules such as HMGB1 and the overt pro-inflammatory cytokines TNF-α and IL-1 [20,35,36,37]. Failure to clear cell debris is known to cause cytokine-mediated induction of osteoclasts [38,39]. The capsules and interface membranes of patients with particle-induced osteolysis showed various strongly expressed apoptosis-related pathways, such as the Fas receptor, BAK, and caspase-3 cleaved [39]. Jersmann et al. showed that fetuin-A leads to increased opsonization of apoptotic cells and macropinocytosis by human macrophages [40]. The authors of this study concluded that fetuin-A is an attractive candidate for future therapeutic intervention in inflammatory diseases [40]. Fetuin-A deficiency might lead to a decrease in the opsonization of apoptotic cells leading to pronounced locally restricted inflammation followed by increased calvarial osteolysis.

The critical role of fetuin-A in mineralized tissue remodeling was initially demonstrated in unchallenged homozygous *Ahsg^−/−^* mice. Up to 70% of these mice suffer epiphysiolysis of the distal femur, causing deformed growth plates and foreshortened proximal limb bones [41,42]. The elevated pre-operative OPG levels in the fetuin-A-deficient mice in our study may reflect an overall osteoanabolic state associated with bone healing following the femoral epiphysiolysis required to heal the slipped growth plates. The enhanced osteolysis found in fetuin-A-deficient mice following induced bone damage suggests that the elevated OPG level cannot compensate for the strong pro-inflammatory and osteocatabolic particle-mediated stimulus.

In summary, we found increased osteoclast activity and decreased osteoblast activity following the local UHMWPE particle challenge of fetuin-A-deficient mice.

### 4.4. Correlation between Fetuin-A and Bone Metabolism in Humans: Fetuin-A Is Slightly Possitivly Associated with Areal Bone Mineral Density in Older Adults

While an osteoprotective effect was demonstrated in the present animal study of fetuin-A deficiency as well as in a prior study on fetuin-A substitution, the effects of the fetuin-A background in humans with aseptic loosening has been recently investigated. In a large cohort of 4714 patients, Fink et al. [43] reported a small positive association between fetuin-A and areal bone mineral density in older adults, but they did not find an association between fetuin-A and the risk of a clinical fracture. Further, Steffen et al. demonstrated that a more favorable outcome after proximal femur fracture was associated with higher fetuin-A serum levels and age during hospitalization [44]. We suggest studying the fetuin-A levels in patients with aseptic loosening as well as the possible association of existing functional fetuin-A polymorphisms on aseptic loosening. In case of confirmed low levels of serum fetuin-A in these patients, a fetuin-A substitution to normal serum levels can be considered as a new treatment option in joint arthroplasty. Any side-effects of fetuin-A intake/application should be investigated beforehand.

We report enhanced particle-induced osteolysis in fetuin-A-deficient mice, but our study has limitations. Despite the irrefutable evidence of disturbed mineral metabolism and bone anomalies in fetuin-A-deficient mice [16,17,20,29,41,42,45,46], these results may not apply to humans. Particle-induced osteolysis was analyzed after fourteen days in the calvaria model, while aseptic prosthetic loosening in humans is a process that occurs over many years. As another limitation, the calvaria model is not analogous to the clinical situation since there is no load-bearing implant [47]. Furthermore, we did not analyze the substitution or over-expression of fetuin-A to prove a possible protective effect on particle-induced osteolysis.

## 5. Conclusions

In conclusion, this study further links clinically relevant particle disease to the glycoprotein fetuin-A. We believe that the interaction of fetuin-A with osteoclast function, inflammation, and apoptosis ultimately leads to in increased particle-induced osteolysis in fetuin-A-deficient mice. Thus, reduced fetuin-A serum levels can be a risk factor for particle-induced osteolysis. Further clinical studies are needed to implement these findings. The protective effect of fetuin-A can be an approach for prophylaxis of loosening of orthopedic endoprostheses and treatment of incipient osteolysis to minimize the number of revision surgeries.

## Figures and Tables

**Table 1:** Bone volume of Fetuin-A-deficient mice (*Ahsg^−/−^*) vs. wild-type (*Ahsg^+/+^*) mice under osteolytic conditions (particles) vs. sham-operated animals. Data are presented in mm^3^ as median and interquartiles range (IOR) and as mean ± standard deviation (SD) with *n* = 5 per group.

Bone Volume	*Ahsg^−/−^*		*Ahsg^+/+^*	
[mm^3^]	Particles	Sham	Particles	Sham
Median (IQR)	0.50 (0.02)	0.53 (0.02)	0.52 (0.01)	0.54 (0.03)
Mean ± SD	0.50 ± 0.01	0.53 ± 0.01	0.52 ± 0.01	0.54 ± 0.02

**Table 2:** Quotient of bone volume/tissue volume of Fetuin-A-deficient (*Ahsg*^−/−^) mice vs. wild-type (*Ahsg^+/+^*) mice under osteolytic conditions (particles) vs. sham-operated animals × 10^−2^. Data are presented in mm^3^ as median and interquartiles range (IOR) and as mean ± standard deviation (SD) with *n* = 5 per group.

Bone Volume/ Tissue Volume [×10^−2^]	*Ahsg^−/−^*		*Ahsg^+/+^*	
	Particles	Sham	Particles	Sham
Median (IQR)	5.69 (0.18)	6.02 (0.05)	6.01 (0.15)	6.19 (0.31)
Mean ± SD	5.71 ± 0.09	6.03 ± 0.03	6.00 ± 0.09	6.25 ± 0.17

**Table 3:** Number of osteoclast in fetuin-A-deficient (*Ahsg^−/−^*) mice vs. wild-type (*Ahsg^+/+^*) mice under osteolytic conditions (particles) vs. sham-operated animals. Data are presented as median and interquartiles range (IOR) and as mean ± standard deviation (SD) with *n* = 5 per group.

Osteoclast Number	*Ahsg^−/−^*		*Ahsg^+/+^*	
	Particles	Sham	Particles	Sham
Median (IQR)	14.5 (10.1)	6.3 (2.9)	16.7 (15.5)	9.7 (8.4)
Mean ± SD	13.5 ± 5.3	6.4 ± 1.5	15.5 ± 8.3	9.7 ± 5.3

**Table 4:** Eroded surface area of fetuin-A-deficient (*Ahsg^−/−^*) mice vs. wild-type (*Ahsg^+/+^*) mice under osteolytic conditions (particles) vs. sham-operated animals. Data are presented in mm^2^ as median and interquartiles range (IOR) and as mean ± standard deviation (SD) with *n* = 5 per group.

Eroded Surface Area [mm^2^]	*Ahsg^−/−^*		*Ahsg^+/+^*	
	Particles	Sham	Particles	Sham
Median (IQR)	0.32 (0.02)	0.10 (0.14)	0.12 (0.10)	0.05 (0.04)
Mean ± SD	0.32 ± 0.01	0.11 ± 0.08	0.14 ± 0.06	0.06 ± 0.02

**Table 5:** Bone metabolism parameter in urine and serum at day 0 and at day 14 in Fetuin-A deficient (Ahsg^−/−^) mice vs. wild type (Ahsg^+/+^) mice a day 0 and at day 14 under osteolytic conditions (particles) vs. sham-operated animals. Data are presented as median and interquartils range (IOR) in the upper row and as mean ± standard deviation (SD) in the lower row with *n* = 10 per group at day 10 and *n* = 5 per group at day 14. The respective units were [nmol/mmol] for DPD/Kreatinin, [pg/mL] for RANKL and Osteoprotegerin, [ng/mL] for Osteocalcin and [mmol/L] for Calcium and Phosphate.

Parameter	Day 0 (Baseline) (*n* = 10 per Group)		Day 14 (*n* = 5 per Group)			
	*Ahsg* ^−/−^	*Ahsg* ^+/+^	*Ahsg* ^−/−^		*Ahsg* ^+/+^	
			Particles	Sham	Particles	Sham
DPD/Creatinin	12.4 (2.1) 12.7 ± 1.5	15.5 (4.8) 16.4 ± 3.5	17.9 (6.1) 16.7± 3.5	16.1 15.9 ± 1.2	22.5 (6.7) 23.8 ± 3.5	19.71 (4.21) 19.0 ± 2.2
RANKL	86.5 (60.3)	95.6 (43.9)	122.6	96.2 (15.1)	128.6 (69.9)	146.4 (32.6)
	96.1 ± 31.0	95.4 ± 27.5	122.2 ± 3.2	94.51 ± 7.9	110.1 ± 47.0	138.0 ± 18.6
Osteo-protegerin	2021 (29) 2046 ± 89	1760 (272) 1685 ± 259	2485 2426 ± 438	1950 (467) 1884 ± 275	1593 (316) 1654 ± 199	1653 (309) 1632 ± 189
Osteocalcin	88.7 (54.1)	116.1 (18.8)	90.0	64.7 (46.4)	120.9 (50.9)	64.6 (35.0)
	82.8 ± 29.3	115.7 ± 12.0	104.0 ± 51.5	71.5 ± 29.5	105.7 ± 27.1	63.1 ± 17.7
Alkaline Phosphatase	195.0 (56.1) 186.9 ± 30.2	339.6 (46.3) 340.4 ± 25.4	100.8 101.5 ± 50.8	98.3 (18.5) 94.6 ± 10.7	266.8 (118.0) 263.5 ± 72.1	227.6 (109.8) 253.3 ± 58.9
Calcium	1.69 (0.31) 1.72 ± 0.17	2.27 (0.20) 2.26 ± 0.12	2.95 2.98 ± 0.6	3.0 (0.6) 2.9 ± 0.3	2.8 (0.7) 3.0 ± 0.4	2.9 (0.3) 2.9 ± 0.2
Phosphate	5.0 (0.6) 5.0 ± 0.6	5.1 (1.5) 5.2 ± 0.8	12.1 12.2 ± 1.1	11.6 (6.3) 12.4 ± 3.4	11.6 (2.6) 12.1 ± 1.6	12.0 (2.2) 12.5 ± 1.3

## Data Availability

Data available on request from the authors.
